# Fulminant septic shock due to *Edwardsiella tarda* infection associated with multiple liver abscesses: a case report and review of the literature

**DOI:** 10.1186/s13256-020-02469-8

**Published:** 2020-09-09

**Authors:** Gultakin Hasan Bakirova, Abdulrahman Alharthy, Silvia Corcione, Waleed Tharwat Aletreby, Ahmed Fouad Mady, Francesco Giuseppe De Rosa, Dimitrios Karakitsos

**Affiliations:** 1 Critical Care Department, King Saud Medical City, Riyadh, Saudi Arabia; 2 Department of Medical Sciences, Infectious Diseases, University of Turin, Turin, Italy; 3 Anesthesia Department, Faculty of Medicine, Tanta University, Tanta, Egypt; 4 Department of Critical Care, Keck School of Medicine, University of Southern California, Los Angeles, CA USA

**Keywords:** Case report, *Edwardsiella tarda*, Septic shock, Liver abscess

## Abstract

**Introduction:**

*Edwardsiella tarda* uncommonly infects humans. The usual presentation is mild gastroenteritis, but systemic manifestations may occur. Lethal infections are rarely documented in patients with underlying disorders.

**Case presentation:**

A previously healthy 37-year-old Southeast Asian woman presented to our hospital with recent onset of abdominal pain, fever, and vomiting. Her condition rapidly deteriorated with signs and symptoms of fulminant septic shock; thus, she was intubated, supported with intravenous vasopressors and fluids, and transferred to the intensive care unit. An abdominal computed tomographic scan with contrast revealed multiple liver abscesses. Blood cultures were obtained and computed tomography–guided percutaneous drainage of the liver abscesses with supplementary cultures was performed; thereafter, empirical broad-spectrum antibiotics were initiated. All cultures grew *E. tarda*, whereas an antibiogram showed resistance to broad-spectrum antibiotics and sensitivity to ciprofloxacin and aminoglycosides; thus, the antibiotic regimen was updated accordingly. The patient made an uneventful recovery and was discharged from the intensive care unit 14 days after admission.

**Conclusion:**

*E. tarda* human infection can present as liver abscess and fulminant septic shock. *E. tarda* strains can be resistant to broad-spectrum antibiotics; hence, culture-based antibiotics should be used accordingly. Clinicians should be aware of this rare and potentially lethal infection.

## Introduction

*Edwardsiella tarda* is a gram-negative, facultative anaerobe that is a member of the family Enterobacteriaceae and was first described by Ewing *et al.* in 1965 [1]. Since then, a growing body of literature has reported *E. tarda* isolates particularly related to brackish water and marine ecosystems, including reptiles, amphibians, and fish [2]. *E. tarda* human infections are rare and mainly present as gastroenteritis; however, extraintestinal and systemic infections have been reported that, when present, are a potentially life-threatening condition carrying up to 50% risk of mortality [3]. Such infections include bacteremia; infections of skin, soft tissue, and biliary tract; liver and tubo-ovarian abscesses; and peritonitis, particularly in immunocompromised hosts [4, 5]. We report a case of *E. tarda* infection that presented as multiple liver abscesses leading to fulminant septic shock in a previously healthy 37-year-old woman.

## Case presentation

**Fig. 1:**
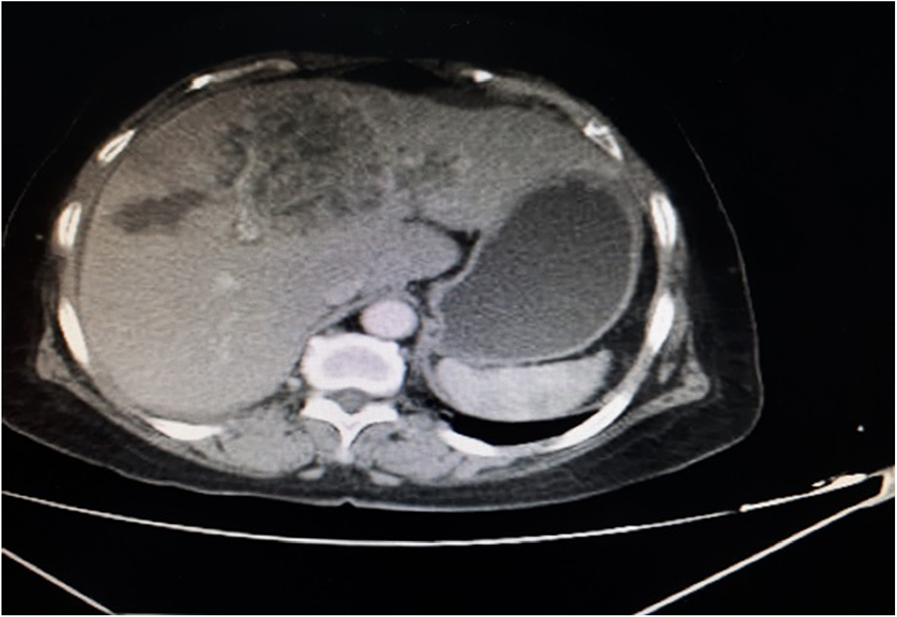
Abdominal computed tomographic scan with contrast revealing liver abscesses (*black arrows*) due to *Edwardsiella tarda* infection

A 37-year-old Southeast Asian woman presented to the emergency department of our hospital with a 5-day history of abdominal pain, fever, and vomiting. Her past medical history was unremarkable apart from a laparoscopic cholecystectomy 10 months earlier. Upon presentation, the patient had high-grade fever, tachycardia, and hypotension suggestive of worsening sepsis, which eventually progressed rapidly to septic shock requiring high doses of noradrenaline and vasopressin. She was intubated, mechanically ventilated, and admitted to the intensive care unit (ICU) accordingly. Upon ICU admission, an abdominal computed tomographic (CT) scan with contrast (Fig. 1) revealed hypodense lesions occupying the right and left liver lobes with calcification foci suspicious of abscesses as well as mild intra-abdominal free fluid collection. Initial investigations revealed a hemoglobin level of 10.4 g/dl and a white blood cell count of 18.9 × 10^9^/L with 89% neutrophils. Her platelet count was normal. Her blood urea nitrogen was raised at 12.1 mmol/L (normal range 2.5–6.4 mmol/L), but her serum creatinine was within normal limits at 115 IU/L (normal range 71–115 IU/L). Her renal function was completely normalized following administration of intravenous fluids a few hours after ICU admission. The findings of blood film obtained for malarial parasites were negative. Notwithstanding this, liver function tests were remarkable with a total bilirubin of 25.3 μmol/L (direct 13.6 μmol/L, indirect 11.7 μmol/L), albumin 19.5 g/L, globulin 44.9 g/L, alkaline phosphatase 416 U/L, alanine aminotransferase 549 IU/L, aspartate aminotransferase 765 IU/L, prothrombin time 16.5 with international normalized ratio 1.41, activated partial thromboplastin time 39.9 seconds, and glucose 5.5 mmol/L. The patient’s physical examination and chest x-ray were unremarkable. CT-guided percutaneous drainage of the liver abscesses with supplementary cultures was performed while blood cultures were derived accordingly. The analysis of the fluid drained from the liver abscess showed a high white blood cell count (11.8 × 10^3^/μl) with polymorphic predominance (77%) and high bilirubin and lactate dehydrogenase levels. Moreover, a gram-stained smear of the pus aspirated from the liver abscess showed few gram-negative bacilli and necrotic debris. All blood cultures as well as stool samples that were collected for 3 consecutive days grew *E. tarda* [6]. Specifically, blood cultures were obtained under sterile conditions and were processed using BacT/ALERT 3D (bioMérieux, Marcy l’Etoile, France). Strains of *E. tarda* were identified using the MicroScan WalkAway 96si system (Siemens, Erlangen, Germany). We evaluated the drug susceptibility of the pathogen based on clinical breakpoints set by the Clinical and Laboratory Standards Institute using E-test (bioMérieux) [7].

Notably, the results of all other infectious and systemic disease workup were negative. Upon ICU admission, piperacillin-tazobactam was initiated empirically (at a dose of 4.5 g intravenously every 6 hours) because the results of the aforementioned cultures were not available at that time. Forty-eight hours later, due to lack of clinical improvement and worsening shock despite fluid resuscitation, antibiotics were empirically once more upgraded to meropenem (1 g every 8 hours by intravenous infusion over 3 hours) and linezolid (600 mg intravenously every 12 hours). As mentioned in the paragraphs above, all cultures grew *E. tarda*. The antibiogram (available when the patient had already received the upgraded antibiotics for 48 hours) showed, interestingly, that the pathogen was resistant to piperacillin-tazobactam and carbapenems while being sensitive to gentamicin and ciprofloxacin. Hence, the antibiotic regimen was updated to ciprofloxacin (400 mg intravenously every 12 hours for 14 days) and gentamicin (400 mg intravenously once daily for 14 days) on the basis of the pertinent antibiogram. The clinical status of the patient gradually improved, and she was successfully weaned off vasopressors and the mechanical ventilator. She was discharged from the ICU 14 days after admission. Thereafter, she was transferred to the gastroenterology ward, where follow-up endoscopic studies revealed mild nonspecific enteritis that eventually resolved completely, and she was discharged from the hospital free of symptoms and in good health.

## Discussion

*E. tarda*, a member of the Enterobacteriaceae family, belongs to the genus *Edwardsiella* along with *E. hoshinae* and *E. ictaluri*, and is a facultative anaerobic gram-negative bacillus. It has a worldwide distribution especially in water environments, whereas its role as a fish pathogen has long been recognized [8]. Exposure to water environments as well as aquatic animals (reptiles, snakes, turtles, and amphibians), iron overload disorders, and ingestion of contaminated raw fish were previously reported as risk factors for infection [3]. *Aeromonas* species, *Vibrio vulnificus*, and *Mycobacterium marinum* share the same environment and therefore a similar exposure risk to *E. tarda*; moreover, the clinical presentation of the former infections could be similar to *E. tarda* infections. although *M. marinum* infections tend to be generally idle and not associated with systemic manifestations [9]. Humans appear to become uncommonly infected with *E. tarda*. Mild gastroenteritis is the most common manifestation associated with this pathogen, but invasive enterocolitis and serious extraintestinal infections, such as necrotizing fasciitis, sepsis, wound abscess, gynecological infections, peritonitis, and osteomyelitis, have been reported in individuals with underlying systemic diseases such as malignancy, impaired immune status, diabetes mellitus, and hepatobiliary diseases [8–10]. Although gastrointestinal infections are generally self-limited [10], *E. tarda*–related septicemia mortality ranges from 38% to 50%, and it should be considered a life-threatening condition [3, 11]. This suggests that a dysfunctional intestinal barrier and/or immune system impairment related to underlying conditions might play a key role in the bacterial translocation across intestinal epithelium, which could in turn facilitate systemic dissemination [11].

We report a case of a previously healthy woman who developed liver abscesses and septic shock due to *E. tarda* infection. Although our patient had a history of cholecystectomy 10 months earlier, she did not have any history of chronic illnesses and denied any exposure to aquatic environments. All cultures that were derived prior to the empirical administration of antibiotics grew *E. tarda*. We are unaware whether previous cholecystectomy *per se* might be considered as a risk factor for developing *E. tarda* infection. A few case reports have outlined patients with a history of cholecystectomy and *E. tarda* infection, but those were also characterized by major underlying disorders [4, 11]. Moreover, previously published articles reported *E. tarda* infections that presented as liver abscesses and/or bacteremia with variable modalities of management and patient outcomes [3, 5, 11–15].

*In vitro* studies revealed that almost all *E. tarda* isolates are susceptible to a wide range of antibacterial agents (ampicillin, most β-lactam antibiotics, quinolones, chloramphenicol, tetracycline, and aminoglycosides) that are commonly used against gram-negative bacteria [16]. In our patient’s case, for the first time, to the best of our knowledge, resistance to broad-spectrum antibiotics such as carbapenems and piperacillin-tazobactam was clearly documented, whereas the *E. tarda* strain was susceptible to ciprofloxacin and aminoglycosides.

Our patient improved gradually during her ICU hospitalization. She finally survived despite the grave clinical presentation of the infection. Surely, our patient was immunocompetent and had no definitive risk factors for *E. tarda* infection, as previously discussed. Moreover, the patient was discharged from the hospital in good health. She shared her appreciation and approval regarding the received treatment and care. We speculate that her survival may be at least partially attributed to both the effective drainage of the liver abscess and the administration of culture-based specific antibiotic therapy apart from the prompt initiation of supportive ICU care. A currently growing body of literature has established *E. tarda* as an unusual cause of serious infections associated with septic shock, even in immunocompetent hosts; hence, intensivists should be aware of this potentially lethal pathogen.

## Conclusion

*E. tarda* human infections could present as liver abscesses and fulminant septic shock even in immunocompetent patients. *E. tarda* strains could be resistant to broad-spectrum antibiotics; hence, culture-based antibiotics should be used accordingly. Clinicians should be aware of this rare and potentially lethal infection.

## Abbreviations

CT: Computed tomography
ICU: Intensive care unit
